# Pneumococcal within-host diversity during colonization, transmission and treatment

**DOI:** 10.1038/s41564-022-01238-1

**Published:** 2022-10-10

**Authors:** Gerry Tonkin-Hill, Clare Ling, Chrispin Chaguza, Susannah J. Salter, Pattaraporn Hinfonthong, Elissavet Nikolaou, Natalie Tate, Andrzej Pastusiak, Claudia Turner, Claire Chewapreecha, Simon D. W. Frost, Jukka Corander, Nicholas J. Croucher, Paul Turner, Stephen D. Bentley

**Affiliations:** 1 Parasites and Microbes, Wellcome Sanger Institute, Cambridge, UK; 2 Department of Biostatistics, University of Oslo, Blindern, Norway; 3 Shoklo Malaria Research Unit, Mahidol-Oxford Tropical Medicine Research Unit, Faculty of Tropical Medicine, Mahidol University, Mae Sot, Thailand; 4 Centre for Tropical Medicine and Global Health, Nuffield Department of Medicine, University of Oxford, Oxford, UK; 5 Department of Epidemiology of Microbial Diseases, Yale School of Public Health, Yale University, New Haven, CT USA; 6 Department of Veterinary Medicine, University of Cambridge, Cambridge, UK; 7 Department of Clinical Sciences, Liverpool School of Tropical Medicine, Liverpool, UK; 8 Infection and Immunity, Murdoch Children’s Research Institute, Melbourne, Victoria Australia; 9 Department of Microbiology and Immunology, Peter Doherty Institute for Infection and Immunity, University of Melbourne, Melbourne, Victoria Australia; 10 Microsoft Research, Redmond, WA USA; 11 Cambodia-Oxford Medical Research Unit, Angkor Hospital for Children, Siem Reap, Cambodia; 12 Mahidol-Oxford Tropical Medicine Research Unit, Faculty of Tropical Medicine, Mahidol University, Bangkok, Thailand; 13 London School of Hygiene and Tropical Medicine, London, UK; 14 Helsinki Institute for Information Technology HIIT, Department of Mathematics and Statistics, University of Helsinki, Helsinki, Finland; 15 MRC Centre for Global Infectious Disease Analysis, Department of Infectious Disease Epidemiology, Imperial College London, London, UK

**Keywords:** Microbial genetics, Bacterial evolution, Bacterial infection

## Abstract

Characterizing the genetic diversity of pathogens within the host promises to greatly improve surveillance and reconstruction of transmission chains. For bacteria, it also informs our understanding of inter-strain competition and how this shapes the distribution of resistant and sensitive bacteria. Here we study the genetic diversity of *Streptococcus pneumoniae* within 468 infants and 145 of their mothers by deep sequencing whole pneumococcal populations from 3,761 longitudinal nasopharyngeal samples. We demonstrate that deep sequencing has unsurpassed sensitivity for detecting multiple colonization, doubling the rate at which highly invasive serotype 1 bacteria were detected in carriage compared with gold-standard methods. The greater resolution identified an elevated rate of transmission from mothers to their children in the first year of the child’s life. Comprehensive treatment data demonstrated that infants were at an elevated risk of both the acquisition and persistent colonization of a multidrug-resistant bacterium following antimicrobial treatment. Some alleles were enriched after antimicrobial treatment, suggesting that they aided persistence, but generally purifying selection dominated within-host evolution. Rates of co-colonization imply that in the absence of treatment, susceptible lineages outcompeted resistant lineages within the host. These results demonstrate the many benefits of deep sequencing for the genomic surveillance of bacterial pathogens.

## Abstract (web summary)

Longitudinal population deep sequencing of *Streptococcus pneumoniae* sampled from infants and their mothers improves our understanding of the dynamics of colonization, transmission, inter-strain competition and the impact of antibiotic treatment.

## Main

*Streptococcus pneumoniae* is a highly recombinogenic human nasopharyngeal commensal and respiratory pathogen causing high rates of pneumonia, bacteremia and meningitis, particularly in young children and the elderly^1,2^. Individual strains are observed to diversify through point mutation, recombination and mobile element acquisition during nasopharyngeal carriage and disease, affecting antimicrobial resistance, susceptibility to vaccine-induced immunity and the inference of transmission networks^3,4^. Further complexity arises from simultaneous carriage of multiple strains. The coexistence of resistant and sensitive strains, and the re-structuring of populations following vaccine introduction, suggest that within-host competition between strains could be critical in the population dynamics of *S. pneumoniae*^5–7^.

As with many bacterial pathogens, surveillance of *S. pneumoniae* has been revolutionized by large-scale whole-genome sequencing (WGS) efforts, which have greatly enhanced our ability to track antibiotic-resistant and vaccine-evading lineages at the population level^8–10^. However, similar to other bacterial pathogens, genomic surveillance of *S. pneumoniae* typically relies on the analysis of a representative genome generated from a single colony from a patient or carrier. This limits the sensitivity of surveillance, as carriage of multiple distinct pneumococcal lineages is frequent in areas with high prevalence^11,12^.

Previous studies of within-host diversity in bacteria predominantly rely on separately sequencing the genomes of multiple purified colonies isolated from an individual, which incurs substantial time and financial cost^13,14^. Conversely, within-host population deep sequencing (PDS) involves sequencing a pool of hundreds of colonies from a sample producing a high depth sampling of within-host diversity. While this provides a more detailed picture of the genetic diversity within the host^15^, these analyses predominantly focus on laboratory studies^16^, relatively small outbreaks^17^ or clinical isolates taken from symptomatic patients, particularly for bacterial species known to colonize patients with cystic fibrosis or other chronic lung diseases^18,19^.

**Fig. 1:**
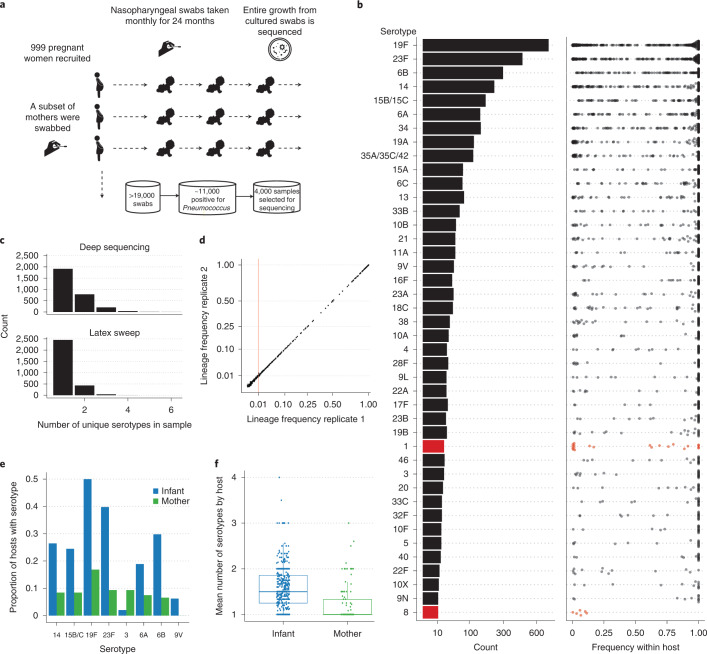
Study design and the frequency of pneumococcal serotypes within the host. **a**, A schematic of the study sampling design. **b**, A barplot indicating the number of times each serotype was observed across all deep-sequenced samples. The distribution of the corresponding within-host frequencies of these serotypes is given in the adjacent plot, with overlapping points separated to indicate the density at each position along the *x* axis. Lineages with ambiguous serotype calls were excluded from this plot. Serotypes found at significantly lower frequencies using the Kolgomorov-Smirnov test are coloured red. **c**, Histograms indicating the distribution of the number of unique serotypes observed using either PDS or latex sweeps. **d**, Comparisons between the estimated GPSC lineage frequencies in 192 samples that were sequenced in replicate. The vertical red line indicates the minimum frequency required for consideration in the mSWEEP pipeline. **e**, Barplots indicating the differences in the representation of serotypes between mothers and infants. **f**, Boxplots indicating the distribution in the mean number of serotypes (excluding non-typables) observed in 107 mothers and 450 of their infants. The median and interquartile range are given by the horizontal lines, with the whiskers indicating the largest and smallest values excluding those outside 1.5 times the interquartile range.

Here, using a deep sequencing approach, we study the evolutionary dynamics of *S. pneumoniae* within healthy carriers, and during episodes of illness and antibiotic treatment, additionally examining the potential utility of within-host population sequencing in surveillance. We analyse data from 3,761 samples collected during a large longitudinal carriage study conducted between 2007 and 2010 in the Maela refugee camp on the border of Thailand and Myanmar^20^. Nasopharyngeal swabs were collected from 965 infants and a subset of their mothers, from birth until 24 months of age (Fig. 1a).

## Results

### Deep sequencing accurately predicts lineage and serotype

We first examined whether accurate lineage and serotype calls could be made from pooled data obtained from deep sequencing hundreds of colonies from plate scrapes of pneumococci grown on selective agar, referred to as PDS. Lineages were defined using the Global Pneumococcal Sequencing Cluster (GPSC) nomenclature^9^. GPSCs consider genome-wide variation to provide a more accurate picture of global pneumococcal population structure. Each GPSC is associated with a small number of serotypes (Supplementary Table 2). Alternatives such as multi-locus sequence typing are limited by the impact of recombination and only consider a small fraction of each genome. Throughout our analyses, we used a dual approach of deconvoluting the mixed samples and running standard analyses, additionally using methods designed for analysing population sequencing data directly (Methods).

We calibrated and verified the approach using a total of 1,210 culture replicates along with a further 192 samples that were sequenced in replicate with separate PCR amplification and library preparation steps. The culture replicates included 1,158 samples for which single colonies had been selected, cultured and sequenced in a previous study and have been re-cultured in this study^21^. In addition, we considered 44 artificial laboratory mixtures for which sequencing data were also available^22^. Finally, a further 8 samples were cultured and deep sequenced in replicate. Of these, only 3 met our initial quality control thresholds for both samples.

**Extended Data Fig. 1:**
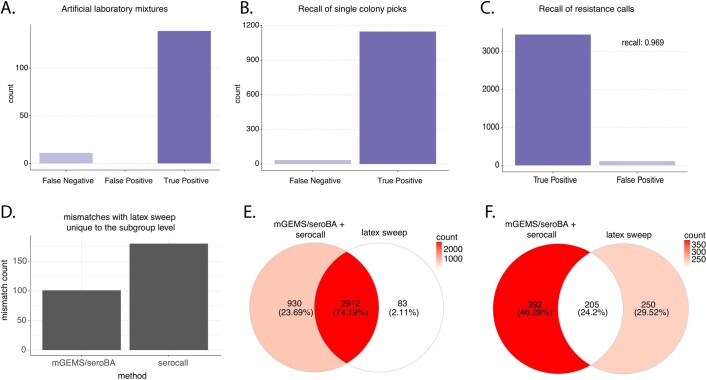
Verification of lineage and serotype calling pipelines. (**a**) The number of true positive and false negative GPSC lineage calls in 44 artificial laboratory mixtures from Knight et al., 2021. (**b**) Recall of GPSC lineage calls in 1158 samples which also had WGS performed on single colony pick in Chewapreecha et al., 2014. (**c**) The recall of resistance calls in the same 1158 samples. (**d**) The number of mismatches between latex sweeps and either mGEMS/seroba or the serocall algorithms at the subgroup serotype level. As mGEMs/seroba was found to better agree with latex sweeps in cases of conflict between the two algorithms, the mGEMs/seroba result was chosen. (**e**) Intersection between the serotype calls of latex sweeps and the combined mGEMs/seroba and serocall pipeline for all typable isolates and (**f**) same as in e. but for non-typable.

The within-host PDS approach reliably detected lineages (GPSCs) in each sample, with a precision and recall of 100% and 93%, respectively, on the artificial laboratory samples indicating that the approach has low false positive rates. It achieved a recall of 97.1% (1,149/1,158) of the lineages present in the larger set of carriage isolates (Extended Data Fig. 1a,b). As only single colony isolates were sequenced, it was impossible to determine the precision in this case. Of the 3 samples that were cultured and deep sequenced in replicate, the approach achieved an accuracy of 100% (3/3). A similarly high accuracy of 97.5% (157/161) was found in the sequencing replicates. Figure 1d shows that the estimated frequency of each lineage was highly concordant between sequencing replicates, with a correlation of >0.99 (*P* < 1 × 10^−3^, Fisher’s *Z*-transform). Although lower, the concordance observed within the three culture replicates (*ρ* = 0.94, *P* = 0.059) was still strong. This indicates that the estimated frequencies are robust to potential artefacts of the experimental pipeline, allowing us to confidently interpret relative changes in frequencies.

### PDS reveals hidden diversity

Using PDS we identified 23.6% (813/3,450) more serotypes compared with the most common method of identifying multiple colonization (latex sweep, Fig. 1c)^11^. Due to difficulties in distinguishing ambiguous or poor-quality serotype calls from non-typables, we assigned such lineages with an ‘unknown’ serotype. Multiple distinct serotypes were observed in 1,028/2,940 (35%) samples, further highlighting the substantial genetic diversity that is obscured by standard surveillance using single representative genomes. The increased sensitivity was supported by microarray data on a subset of 32 samples performed in a previous study, which identified all 49 serotypes found by PDS, compared with 32 found using latex sweeps^11^. Unlike PDS, microarray data only indicate the presence and absence of known genes and serotypes, and do not provide data over the entire genome.

Rates of multiple colonization were significantly higher in infants than in their mothers (*P* < 1 × 10^−3^, Poisson mixed model) (Fig. 1f). The most common serotypes, including 19F and 23F, were also significantly more likely to be found in infants (Fig. 1e), consistent with a greater repertoire of adaptive immunity in adults (adjusted *P* < 0.05, Fisher’s exact test)^23^. In agreement with past studies, serotype 3 was the only serotype likely to be found more frequently in mothers (Fig. 1e)^24,25^. It has been postulated that the high rate of invasive disease due to serotype 3 in adults may correlate with high antibody levels in children, which then wane^26^.

Other ‘epidemic’ serotypes (for example, 1, 2, 5, 7F, 8 and 12F) are known for causing outbreaks of disease in adults despite being rarely detected in infant carriage^27^. Strikingly, we found that such types were often present at low frequencies within the host (Fig. 1b). In particular, serotypes 1 and 8, and the associated GPSCs 2 and 28, were found at lower frequencies than other types (adjusted *P* value <0.05, Kolgomorov-Smirnov test). In 11/20 (55%) observed cases of serotype 1 in our dataset, it was found as the minority serotype in multiple colonization. This could partly explain its low detection rate in previous carriage studies^9^, which typically only detect each sample’s dominant strain. Given that invasiveness is usually calculated by comparing carriage and disease rates, this suggests that current estimates of the invasiveness of serotype 1 may be inflated. However, despite PDS identifying over twice as many serotype 1 lineages, the overall prevalence of this serotype was still low, making up <1% of all distinct serotype-host pairs in the dataset. Nevertheless, this serotype still appears to be highly invasive, justifying its targeting by current vaccines^28^.

**Extended Data Fig. 2:**
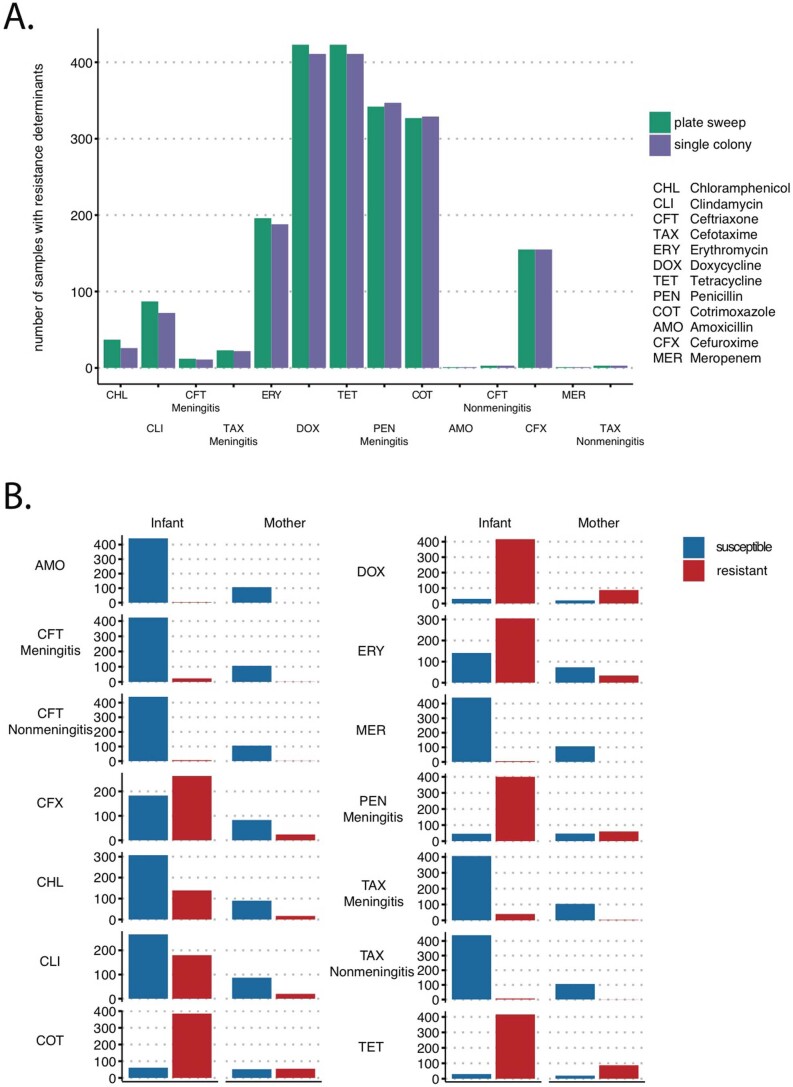
Verification of resistance calling pipeline as well as the distribution of resistance calls in mothers and infants. (**a**) The number of resistance calls identified in 584 samples which consisted of only a single pneumococcal lineage and were sequenced using PDS and via single colony picks in Chewapreecha et al., 2014. The high correspondence between the two methods suggests PDS has a low false positive rate. (**b**) The number of samples found to be either resistant or susceptible to each antibiotic class for both mothers and infants. Resistance was determined by running the CDC pneumococcal resistance pipeline on the deconvoluted lineages output by the mGEMS pipeline. The individual lineage calls were collapsed to the sample level so that a sample was called as ‘resistant’ if resistance was observed in any of its lineage.

We found that PDS identified an additional 14.6% (520/3,557) of resistance elements, including known resistance single nucleotide polymorphisms (SNPs) and mobile genetic elements, when compared with using standard pipelines on the set of 1,158 single-colony whole-genome sequences (Extended Data Fig. 1c). Resistant lineages were frequently found alongside susceptible lineages within the same host. The rate of resistance in samples taken from infants was significantly higher than that in mothers for 4/14 antibiotic classes, which corresponds with the difference in the composition of lineages observed between mothers and children (Extended Data Fig. 2b, adjusted *P* < 0.05). Thus, routine PDS provides substantial improvements over alternative approaches in surveillance of pneumococcal resistance, especially in children where rates of multiple colonization are higher.

### Within-host diversity provides insights into transmission

**Fig. 2:**
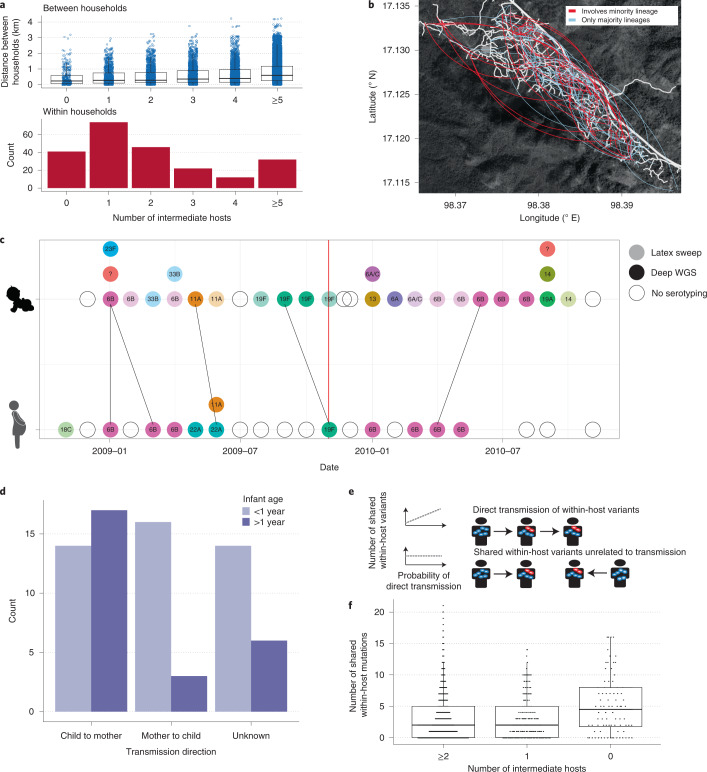
Transmission dynamics within the Maela refugee camp. **a**, Top: the distribution of pairwise geographic distances between 411 different households versus the number of intermediate transmission events as inferred using the modified TransCluster algorithm. The median and interquartile range are given by the horizontal lines, with the whiskers indicating the largest and smallest values excluding those outside 1.5 times the interquartile range. Bottom: the distribution of estimated intermediate transmission events within households. **b**, A map of the Maela refugee camp, with inferred direct transmission links overlaid. Roads are shown in white. The direction of transmission is not estimated. Blue lines indicate transmission links that would typically be inferred using a representative genome per sample, while red lines indicate additional links that were found using PDS. **c**, A representative mother-child pair indicating how transmission direction was inferred. Coloured circles indicate the serotypes present, with PDS data available for those coloured in darker shade. Black lines indicate close transmission links inferred using the TransCluster algorithm, with the vertical red line indicating the time the child was one-year old. **d**, The distribution of the direction of transmission between mother and child split by whether the transmission event occurred before or after the child turned one. **e**, A schematic demonstrating that we would expect to see an elevated rate of polymorphic sites (represented by blue and red variants) among close transmission pairs. **f**, The distribution of the number of shared polymorphic sites in 3,663 potential transmission pairs involving an estimated 0, 1 or ≥2 intermediate hosts. The elevated number of variants involving 0 intermediates hosts indicates a mean bottleneck size ≥1. The median and interquartile range are given by the horizontal lines, with the whiskers indicating the largest and smallest values excluding those outside 1.5 times the interquartile range.

Deep within-host population sequencing also allows for improved estimates of transmission links^3,29^. To provide a robust measure of the strength of a transmission link between any two samples in our dataset, we adapted the TransCluster algorithm to account for within-host diversity information (Methods)^30^. There was a strong association between the probability of direct transmission, as inferred by the adapted TransCluster algorithm independently of location data, and the geographic proximity of households (*P* < 1 × 10^−3^, linear model *t*-test; Fig. 2a,b). This association remained after excluding within-household pairs involving mothers and their children (*P* < 1 × 10^−3^), suggesting that children living closer to detected cases of more invasive strains are at higher risk, which could motivate local interventions to reduce transmission in outbreaks. Of the inferred close transmission links (estimated to involve either 0 or 1 intermediate hosts), 80.9% (871/1,077) contained at least one sample found to carry multiple pneumococcal lineages. This can be partly attributed to the high level of multiple colonization in the cohort, but nevertheless suggests that only considering the dominant lineage will substantially underestimate the number of close transmission links.

This high-resolution approach also allowed us to scrutinize the transmission bottleneck, which is the point of the pneumococcal lifecycle blocked by immunity induced by current vaccines^31^. Laboratory experiments have indicated that there is a very tight bottleneck in the transmission of *S. pneumoniae*, consisting of only a single bacterial cell^32^. To understand how well these experiments generalize to transmission in human hosts, we took a conservative approach, using only samples containing a single strain (Methods). The substantial increase in the number of shared polymorphic sites found in putative direct transmission pairs relative to those estimated to involve intermediate hosts suggests that while tight, the transmission bottleneck between the donor and recipient is probably greater than one (*P* < 1 × 10^−3^, Poisson regression) (Fig. 2e,f). Mouse models of pneumococcal transmission have indicated that this bottleneck is likely to occur following exit but before establishment in the recipient host^32^.

To examine transmission within the home, we next considered the 47 mother-child pairs for which a transmission link involving zero or one intermediate host was inferred using the TransCluster algorithm. To estimate a plausible direction of transmission, we required that the infector must have acquired the relevant lineage before the infectee, and that there could be at most one negative or missing sample in the infector in the 2 months before the infectee becoming infected with the same lineage (Fig. 2c). The vast majority (16/19) of mother to child transmissions occurred in the first year of the infant’s life (Fig. 2d). This was significantly different from the child to mother transmissions (14/31, *P* = 0.008 Fisher’s exact test). This difference remained after excluding transmission events in the first two months of the infant’s life allowing additional time for colonization to occur (12/15, *P* = 0.031). The observed asymmetry is consistent with <1-year-old infants being more susceptible to infections from within the household, and with the high proximity between mother and child. The exposure risk posed by adults has been observed in previous studies^33,34^, with routine vaccination of older children not found to have a significant effect on vaccine type carriage rates in unvaccinated infants^35^. Taken together, this suggests a possible benefit to a vaccination campaign targeting mothers or other adults with high contact rates to young infants before herd immunity in the adult population is established. However, this would not reduce the risk posed by non-vaccine type lineages.

### Strong purifying selection and a unique mutational spectrum

**Fig. 3:**
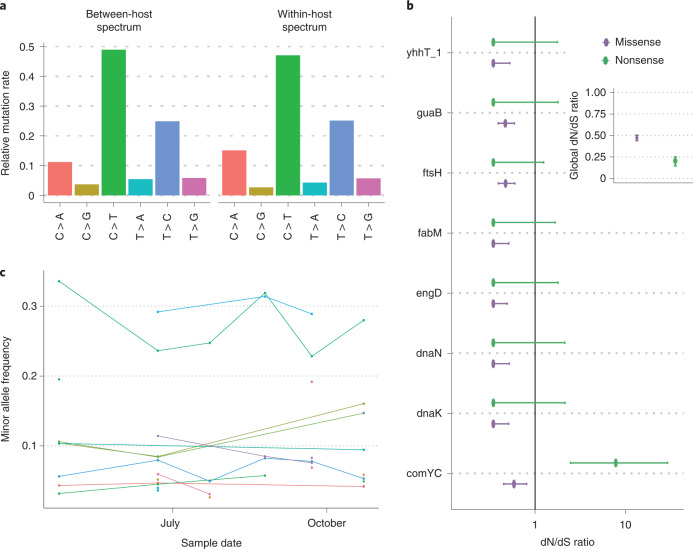
Mutational spectra and selection within the host. **a**, The relative fraction of different single-nucleotide base changes found within the host in 1,627 samples involving only a single pneumococcal lineage compared to those changes observed between hosts inferred using ancestral state reconstruction. **b**, dN/dS ratios for genes found to be under significant selection (adjusted *P* < 0.05) within the host in 1,592 samples using the Poisson individual gene model in the dNdScv R package. Error bars indicate the 95% confidence intervals of the coefficient in the regression. The grey line indicates a dN/dS ratio of one, indicating the separation of positive and negative selection. **c**, An example of the within-host variant allele frequencies over five consecutive samples, taken from a single infant colonized with a single pneumococcal lineage (GPSC 47), which is not a common ‘epidemic’ lineage. Each coloured line indicates the frequency of a different within-host variant.

**Extended Data Fig. 3:**
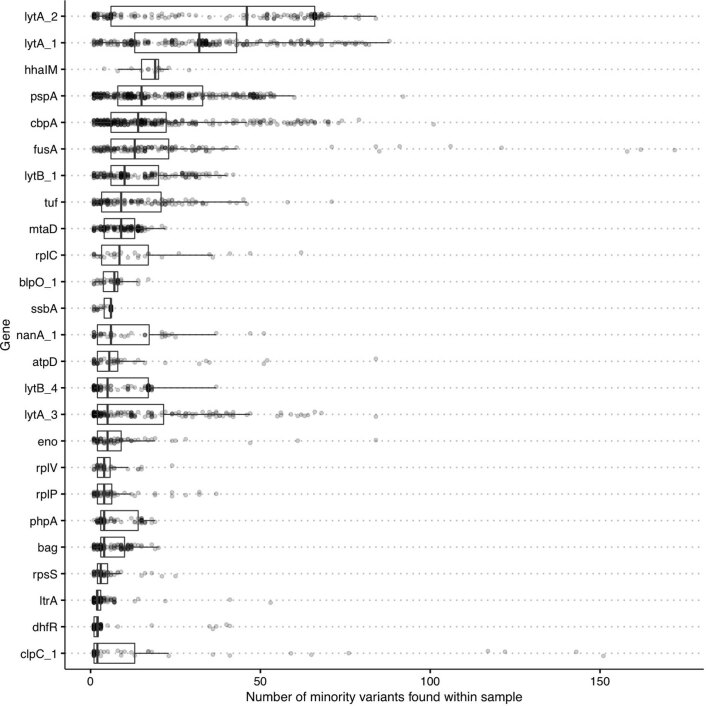
Distribution of SNV found in regions with elevated rates of polymorphisms. Boxplots indicating the distribution of the number of SNV found in regions with elevated rates of polymorphism from 1592 samples classified by the spatial scan statistic (see Methods). The median and interquartile range is given by the horizontal lines with the whiskers indicating the largest and smallest values excluding those outside 1.5 times the interquartile range. The high rate of polymorphisms in these region indicates that these SNVs are unlikely to be the result of denovo mutation within the host and are instead likely to be driven by recombination, gene duplication, homology with phages and co-colonising bacterial species and hard to align regions.

To investigate selection acting at the scale of individual lineages within the host we restricted our analysis to within-host single nucleotide variants (SNVs) found in samples involving only a single pneumococcal lineage (Fig. 3c). This avoided the potential for biases or errors being introduced by the deconvolution of mixed samples. Minority variants were called using a conservative pipeline that included a scan statistic to filter out regions likely to be affected by homologous recombination, gene duplications and similarity with bacteriophages and other bacterial species (Methods). Many of the regions identified by this scan included genes coding for major pneumococcal autolysin proteins (including LytA) and other surface-associated choline binding proteins (CBP, including pneumococcal surface proteins A and C, PspA and PspC) and the Tuf elongation factor (Extended Data Fig. 3). Homologues to LytA and CBP domains are frequently found in pneumococcal phages or co-colonizing bacterial species, which may facilitate pneumococcal diversification and recombination in these regions^36,37^.

**Extended Data Fig. 4:**
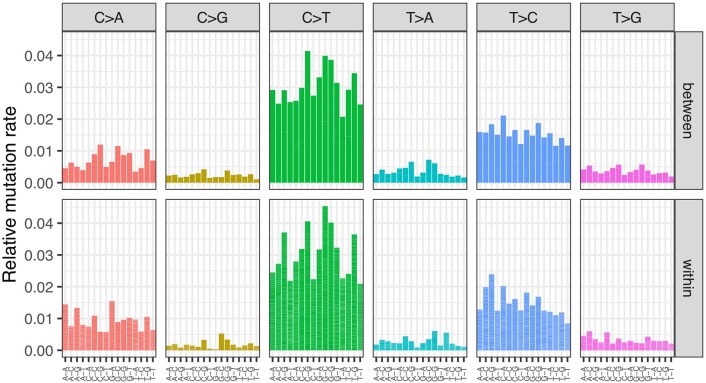
Within and between host mutational spectra for each of 96 tri nucleotide substitution classes. Each spectra is displayed according to the 96 substitution classification defined by the substitution class (colour in the graph) and sequence context immediately 3′ and 5′ to the mutated base. The mutation types are given on the horizontal axes, while vertical axes depict the frequency of each type. Mutations observed using ancestral state reconstruction from genomes observed in different hosts are given above while mutations observed within a host are given in the bottom panels.

The remaining within-host single nucleotide variants displayed a mutational spectrum similar to that found in the genome phylogenies constructed from single colonies taken from separate hosts (Fig. 3a and Extended Data Fig. 4)^21^. This indicates that similar mutational processes act across the different timescales. Although the spectra were similar, we observed elevated numbers of C→A transversions with weak sequence context in the deep-sequencing calls (*P* < 1 × 10^−3^, permutation test). This is consistent with oxidative- and deamination-induced damage, which is typically reduced in frequency by purifying selection over longer timescales^38^. A similar enrichment of C→A mutation was found in *E. coli* over short timescales, which may be driven by the misincorporation of adenines into cytosine sites^39^. Finally, pneumococci carry the *spxB* gene that secretes hydrogen peroxide and has been shown to cause DNA damage to host lung cells and may contribute to the mutational spectrum of the bacterium itself.

To investigate signatures of selection, we calculated dN/dS ratios using a modified version of the dNdScv package^40^. Similar to other respiratory pathogens, we found a strong signal of purifying selection, particularly against nonsense mutations (Fig. 3b)^13,19,41^. This was also observed at the level of individual genes, with only the competence related gene (comYC) having an elevated rate of nonsense mutations (Benjamini–Hochberg adjusted *P* < 0.05) (Fig. 3b). The frequent insertion of pneumococcal prophage into comYC causes premature stop codons that prevent the host cell from undergoing transformation and are associated with a reduced duration of carriage^42–44^.

The strongest evidence of purifying selection was observed in genes associated with the pneumococcal stress response, including heat-shock proteins dnaK and ftsH, as well as fabM which is necessary for survival in high-acidity environments^45^. Multiple-antigen *S. pneumoniae* vaccines which include DnaK, as well as other heat-shock proteins, have been shown to protect against lethal pneumococcal challenge^46^. FabM has also been suggested as a potential target for novel chemotherapeutic agents^47^. The observed purifying selection indicates that it may be difficult for the pneumococcus to adapt to treatments targeting these genes over short timescales. Although we were able to detect purifying selection, using dN/dS we did not find evidence for short-term adaptive evolution in any genes. This probably reflects the long-term commensal lifestyle of *S. pneumoniae* in contrast to that seen in environmental or immunocompromised patient pathogens^18,19^.

### Within-host competition between pneumococcal lineages

**Extended Data Fig. 5:**
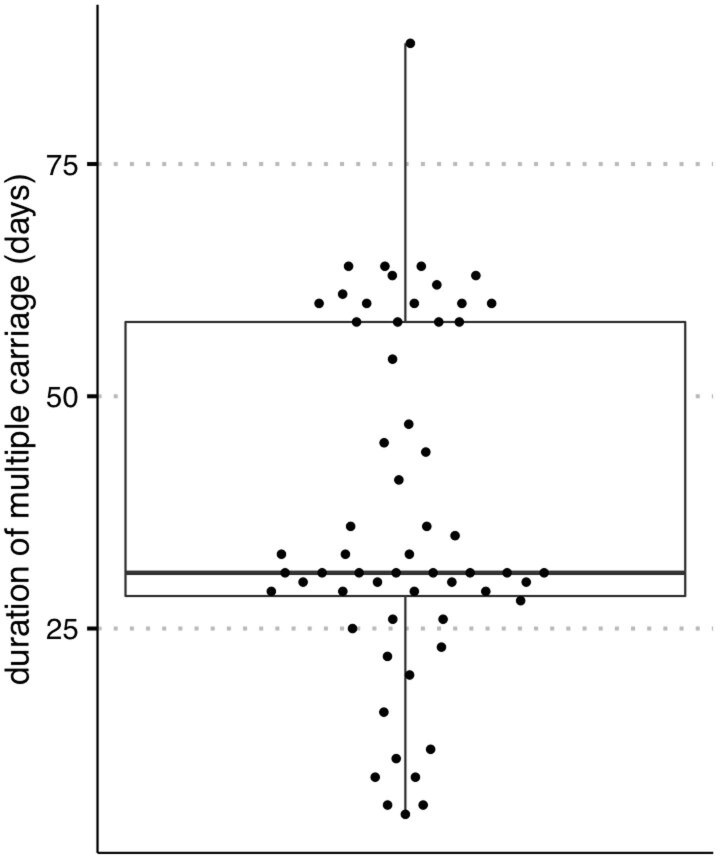
Distribution of multiple carriage duration. The distribution of the length of time multiple lineages colonised the same host in one of 59 multiple carriage events where the same lineages were observed in consecutive samples. Multiple colonisation events that were only observed at a single time point are excluded from this analysis. The median and interquartile range is given by the horizontal lines with the whiskers indicating the largest and smallest values excluding those outside 1.5 times the interquartile range.

The majority of multiple colonization events between different GPSCs were observed at only a single timepoint in 92.3% (712/771) of events, indicating that long-term multiple colonization of the same lineages is rare. However, we did observe a number of carriage events where two lineages coexisted for well over the month-long time period between routine sampling. This suggests that competition between lineages within the host is not always strong enough for one to exclude the other (Extended Data Fig. 5). Despite the large sample size, we did not have the statistical power to identify any preferential co-colonization between particular pneumococcal lineages due to the high number of possible combinations.

**Fig. 4:**
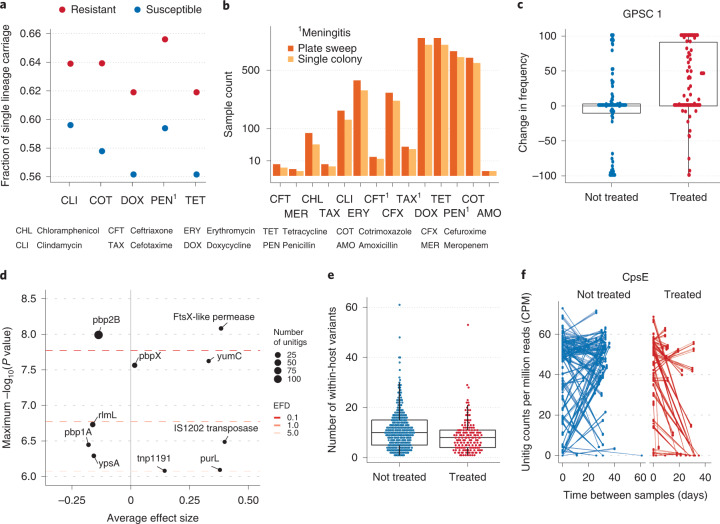
Within-host dynamics of antimicrobial resistance and the impact of antibiotic treatment. **a**, The fraction of carriage events consisting of a single lineage found to be resistant to each antibiotic class. Only those classes found to be less likely to occur in instances of multiple colonization than expected given the background prevalence in the population are shown. **b**, The number of resistance calls for each antibiotic class in 1,158 samples for which both single colony picks and PDS had been performed. **c**, The distribution of the change in frequency of the GPSC1 lineage in 182 pairs of consecutive samples that have and have not received antimicrobial treatment. The median and interquartile range are given by the horizontal lines, with the whiskers indicating the largest and smallest values excluding those outside 1.5 times the interquartile range. **d**, A dot plot indicating the significance and effect size of unitigs found to be associated with antimicrobial treatment using a linear mixed model in Pyseer. **e**, The number of within-host SNV in 1,192 samples taken from distinct carriage episodes involving only a single pneumococcal lineage split by recently received antimicrobial treatment. **f**, The normalized count of unitigs found in CpsE in pairs of samples where a subset had received treatment between sampling events.

While resistant lineages were frequently observed to co-colonize with susceptible lineages, this occurred less frequently than expected given the frequency of resistant lineages within the Maela camp (Fig. 4a). We found that rates of resistance in multiple colonization were significantly lower than expected in 5/14 antibiotic classes, including penicillin, indicating that susceptible lineages outcompete resistant lineages within the host^48^. Many models of the maintenance of antibiotic resistance in pneumococcal populations rely on assumptions about the competition between resistant and susceptible lineages^5,6,49^. However, studies have currently relied on serotype data alone to determine multiple colonization rates, which do not indicate whether the underlying lineages are resistant to antibiotics. This result confirms that resistant and susceptible lineages are found to co-colonize the same host, and that the expected fitness costs of resistance observed in laboratory experiments are consistent with the population dynamics observed in natural pneumococcal carriage.

### Strong impact of treatment on within-host diversity

**Extended Data Fig. 6:**
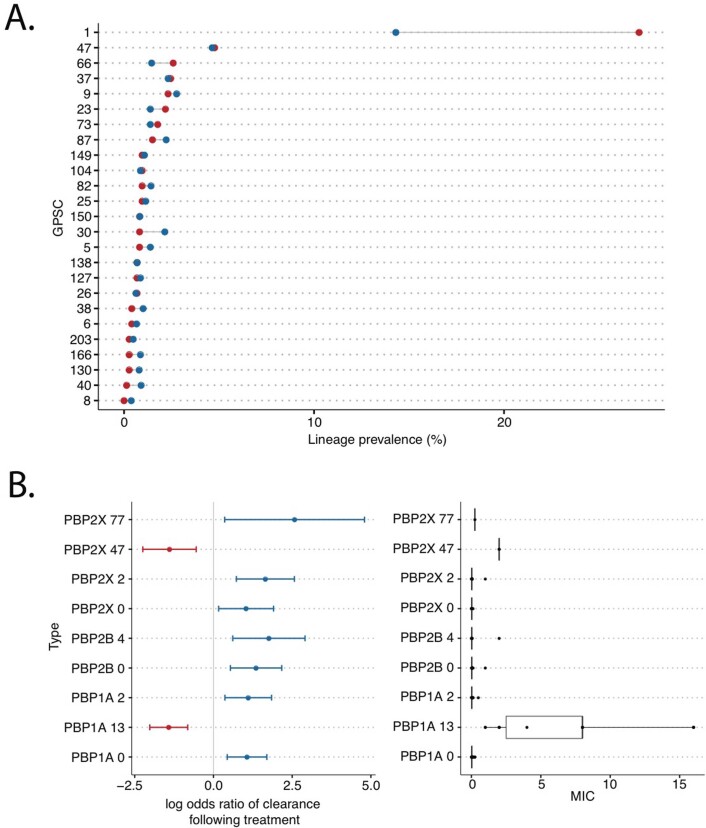
Impact of antimicrobial treatment on GPSCs and lineages classified by PBP gene type of Li et al., 2016. (**a**) The proportion of lineages made up of each GPSC after treatment (red) and in the absence of treatment (blue). Only those GPSCs that are present at a prevalence of at least 1% in the full data set are included. (**b**) The log odds ratio of clearance following treatment for 1,848 lineages containing previously classified PBP genes in Li et al., 2016 (left). Those in red are significantly more likely to persist following antimicrobial treatment whilst those in blue are likely to be eliminated. Error bars indicate the standard deviation of the coefficient point estimate in the GLM. The corresponding distribution of MIC profiles for lineages found to contain these PBP genes in Li et al. is given to the right. The median and interquartile range is given by the horizontal lines with the whiskers indicating the largest and smallest values excluding those outside 1.5 times the interquartile range.

We next considered selection in response to antimicrobial treatment, both in terms of the displacement of pre-treatment strains and the microevolution of surviving pneumococci. Pairs of consecutive samples taken from the same infants within 100 d were selected, where a subset had received antibiotics between the sampling timepoints. GPSC1 was found at considerably higher frequencies than other GPSCs following treatment (Fig. 4c and Extended Data Fig. 6a). GPSC1 lineage is a known multidrug-resistant (MDR) lineage with a pre-dominant predicted MDR antibiogram of penicillin, cotrimoxazole, erythromycin and tetracycline resistance^9^. A similar analysis using the penicillin binding protein (PBP) gene ‘types’ used in the in-silico classification of pneumococcal resistance by the US Centers for Disease Control and Prevention (CDC)^50^ identified the *pbp2X*-47 and *pbp1A*-13 types as being strongly associated with the persistence of a lineage post treatment (Extended Data Fig. 6b). These are the most common types in GPSC1 in Maela, representing 54% and 98% of the single colony isolates, respectively^21^. Alterations in the PBPs reduce their affinity for penicillin and thus susceptibility to beta-lactam antibiotics while allowing them to maintain their role in cell wall metabolism.

Although it has been suggested that the increase in resistant isolates following treatment is due to the elimination of susceptible lineages^51^, we found that this pattern is observed after controlling for the presence of GPSC1 in the first sample of a pair. This suggests that treatment increases the frequency of GPSC1 by both eliminating competing lineages and reducing competition for colonizing resistant strains. This could motivate pre-emptive interventions, such as limiting contacts with high-risk individuals following antibiotic treatment.

**Extended Data Fig. 7:**
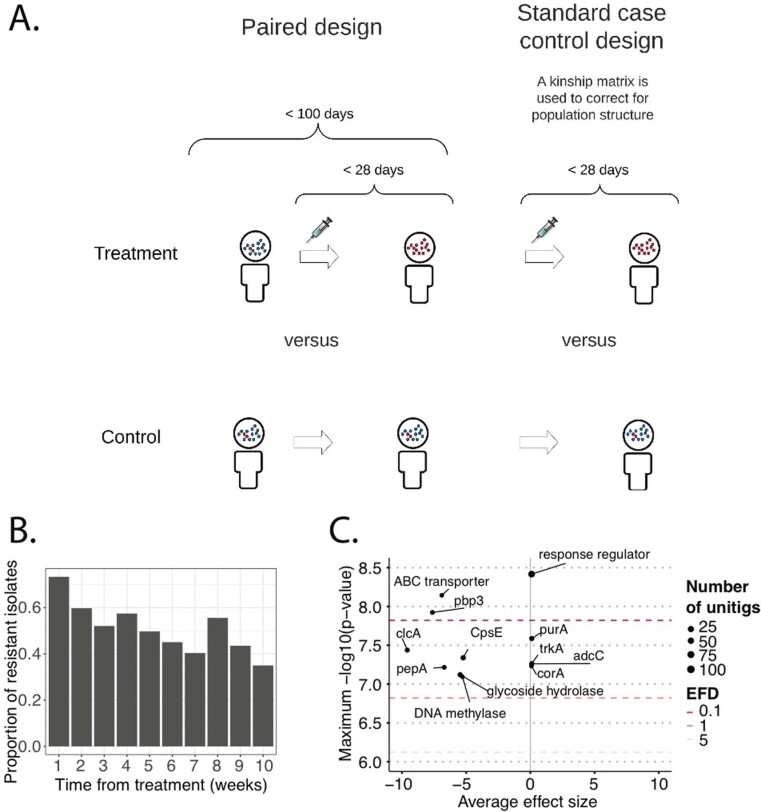
GWAS study design and results of paired sample analysis. (**a**) A schematic indicating the design of the two GWAS analyses conducted in this study with red and blue points indicating within-host polymorphisms. A linear model on the log of the unitig counts per million similar to that commonly used in RNA-seq analyses was used in the paired design while the Pyseer algorithm was used in the standard design. (**b**) The proportion of resistant isolates following antimicrobial treatment. Those samples within a threshold of 4 weeks (28 days) of a treatment event were classified into the ‘treated’ class. (**c**) A dot plot indicating the significance and average effect size of unitigs found to be associated with treatment in the analysis of paired samples taken from the same host where a subset have received antimicrobial treatment in between sampling events. Regressions were performed using a linear model with the frequency of the unitig within the host taken as the dependent variable (Methods). The horizontal red lines indicate the expected number of false discoveries (EFD) providing different significance levels to interpret the resulting variant calls.

To investigate selection acting within a single carriage event, we considered loci within the set of paired samples where the same lineage was present in both samples of a pair (Methods). This revealed that the diversity of within-host variants reduced markedly following antimicrobial treatment (Fig. 4e). A number of variants were found at lower frequencies post treatment including in the capsular gene cpsE (Fig. 4f and Extended Data Fig. 7c). Point mutations in cpsE have been shown to alter the growth, adherence and competence of pneumococci^52^. Common variants with smaller effect sizes were observed in the adenylosuccinate synthetase gene (purA), and genes involved in the zinc (adcC) and magnesium transport (corA) systems, which have all been observed to be downregulated in response to sub-inhibitory concentrations of penicillin^53^. Taken together, antimicrobial treatment produces a strong bottleneck within the host even when the resident strain is resistant. The generation of low-frequency variants that are then eliminated after treatment may be an example of short-sighted evolution^54^.

While the paired-sample deep population genome-wide association study (GWAS) can identify changes occurring within a single carriage event, it is unable to identify variation associated with selection against whole lineages. By comparing the presence and absence of unitigs in samples taken within 28 d of treatment to those that had not been treated, we identified a number of sequence elements associated with antimicrobial treatment (Fig. 4d). This included elements found in *pbp2B*, *pbp2X* and *pbp1A*—three of the genes that encode for the major penicillin binding proteins which are critical in determining non-susceptibility to beta-lactam antibiotics^55^. Interestingly, the strongest association was with *pbp2B*, which is the primary gene for low-level penicillin resistance, and is consistent with amoxicillin being used for treatment in the majority (66.9%) of cases^56^. The stronger association with *pbp2B* indicates that resistance conferred by these mutations is found across a diverse set of lineages, while the associations observed in the paired analysis of PBP types are driven primarily by particular lineages such as GPSC1.

We also observed associations with the membrane protein FtsX, a ribosomal RNA methyltransferase (rlmL) and a ligand binding protein (YpsA). FtsX is involved in cell division and is thought to co-locate with both *pbp2b* and *pbp2x* in the outer-ring peripheral peptidoglycan synthesis machine during cell division^57^. YpsA is also linked to pneumococcal cell division^58^. RmlL is thought to facilitate resistance occurring through other mutations^59^. Variation at these loci could allow pneumococci to slow down their metabolism and cell division, increasing the population’s chances of persisting over the time period when the antibiotic is present. We also observed a weak association with the insertion sequence IS1202, which has been closely linked to the MDR-associated serotype 19F and its capsular polysaccharide synthesis (cps) locus, which is predominantly found in GPSC1^60^.

## Discussion

Our ability to understand the within-host evolution and transmission of *S. pneumoniae* is essential to developing successful public health interventions. We have shown that deep within-host population sequencing can lead to substantial improvements in surveillance of high-risk genotypes, reconstruction of transmission chains, and understanding the impact of antibiotic resistance on co-colonization and competition. In particular, we were able to double our sensitivity for detecting the highly invasive serotype 1 in carriage. These lineages were often found at low frequencies, which may explain the disconnect between their high prevalence in invasive disease and scarcity in carriage studies that rely on either latex sweeps or representative genomes. The increased resolution of PDS also revealed an age-dependent rate of transmission between mothers and infants. This, coupled with the strong association between geographic distance and the likelihood of direct transmission within the Maela refugee camp, suggests that interventions targeting close contacts could be particularly important for reducing disease and colonization by resistant lineages in early childhood before vaccination and following antimicrobial treatment.

These results demonstrate the substantial improvement PDS can provide in the near-to-real time surveillance of pathogens with high rates of multiple colonization. In such pathogens, ignoring within-host diversity can lead to a substantial fraction of colonization and transmission events being missed. The implementation of PDS in routine surveillance would require procedures very similar to those currently used in public health laboratories that make use of WGS. The initial culture step remains the same, with the main change being the depth of sequencing. This is rapidly becoming more affordable. However, the computational analysis of these data is substantially more complicated, which currently limits this surveillance to laboratories with advanced genomic analytics capabilities. This is likely to improve as analytical methods become more robust and easier to use.

Our results provide a large-scale dataset on the natural co-colonization of both resistant and susceptible pneumococcal lineages within the same host. We provide clear evidence that such coexistence is frequent (previously an assumption made by a number of models^5,6,49^) and find that resistant lineages appear less often than expected in multiple colonization given their overall frequency within the population, consistent with the lower fitness of resistant lineages observed in laboratory experiments. The negative association between resistance and multiple colonization, combined with the association between antimicrobial treatment and subsequent colonization by a multidrug-resistant strain, indicates that reduced within-host competition following treatment plays a major role in the risk of an infant being colonized by an MDR lineage. This emphasizes that the broader dynamics of pathogen population structure and inter-strain competition must be a key consideration in the design of vaccines and other interventions^28^. The observed competition could also motivate the use of pre-emptive probiotics to protect against colonization by more dangerous lineages, although trials of such approaches have returned mixed results^61,62^. The strong negative selection observed in heat-shock proteins suggests that multiple-antigen vaccines may provide a valuable alternative to current capsule-specific vaccines as they have the potential to elicit cross-serotype protection^46^. Overall, the added insights into selection and evolution within the host, coupled with the substantial improvements in transmission inference and surveillance, present a compelling case for the future routine use of deep within-host population sequencing in the research and surveillance of common bacterial pathogens.

## Methods

### Sample selection

Nasopharyngeal swabs were collected between November 2007 and November 2010 from an initial cohort of 999 pregnant women, leading to the enrolment of 965 infants as part of a previous study^20^. Ethical approval for the original study was overseen by the Faculty of Tropical Medicine, Mahidol University, Thailand (MUTM-2009-306) and Oxford University, UK (OXTREC-031-06). Swabs were taken monthly from birth for the first 24 months of the infant’s life. Of the original cohort, swabs were obtained from 952 mothers, with dropouts largely due to intrauterine deaths in the 3rd trimester and stillbirths. In total, 636 infants completed the full 24 months of the study, with the majority of those lost having left the camp. The outcome of the full cohort is given in the supplementary data available on GitHub. A total of 23,910 swabs were collected during the original cohort study, including 19,359 swabs that were processed according to World Health Organization (WHO) pneumococcal carriage detection protocols^63^ and/or the latex sweep method^64^. All isolates were serotyped using latex agglutination as previously described^11^. In addition to swabs, the household location, episodes of infant illness and antibiotic treatment were all recorded over the 24-month sampling period for each infant.

Deep sequencing of sweeps of colonies was attempted on a subset of 4,000 swabs. All swabs taken before and after an antibiotic treatment event were selected. Further swabs were included if they were inferred to be within close transmission links corresponding to a distance of <10 SNPs, using a previously sequenced set of 3,085 whole-genome sequences obtained from single-colony picks^21^. This allowed for increased resolution into both the impact of antibiotic treatment on within-host diversity and consideration of the transmission bottleneck. A subset of 25 mother-child pairs were also sequenced at a higher temporal resolution of at least once every 2 months. These mother-child pairs were chosen if they had completed the full 24 months of the study and if a number of samples had already been selected for sequencing in the first two sample selection steps. The remaining samples were selected randomly.

### Culture and sequencing

Nasopharyngeal swabs (100 μl) stored at −80 °C in skim milk, tryptone, glucose and glycerine media were plated onto Columbia CNA agar containing 5% sheep blood (BioMerieux, 43071). These were incubated overnight at 37 ± 2 °C with 5% CO_2_. All growth was collected using sterile plastic loops and placed directly into Wizard genomic DNA purification kit nuclei lysis solution (Promega, A1120). The Wizard kit extraction protocol was then followed, eluting in 100 μl of the provided DNA rehydration solution. DNA was quantified with a BioPhotometer D30 (Eppendorf) and then stored at −80 °C before sequencing. DNA extractions were sequenced if they contained more than 2.5 μg of DNA. Sequencing was performed at the Wellcome Sanger Institute on an Illumina NovaSeq at 192 plex using unique dual index tag sets.

### Quality control filtering

**Extended Data Fig. 8:**
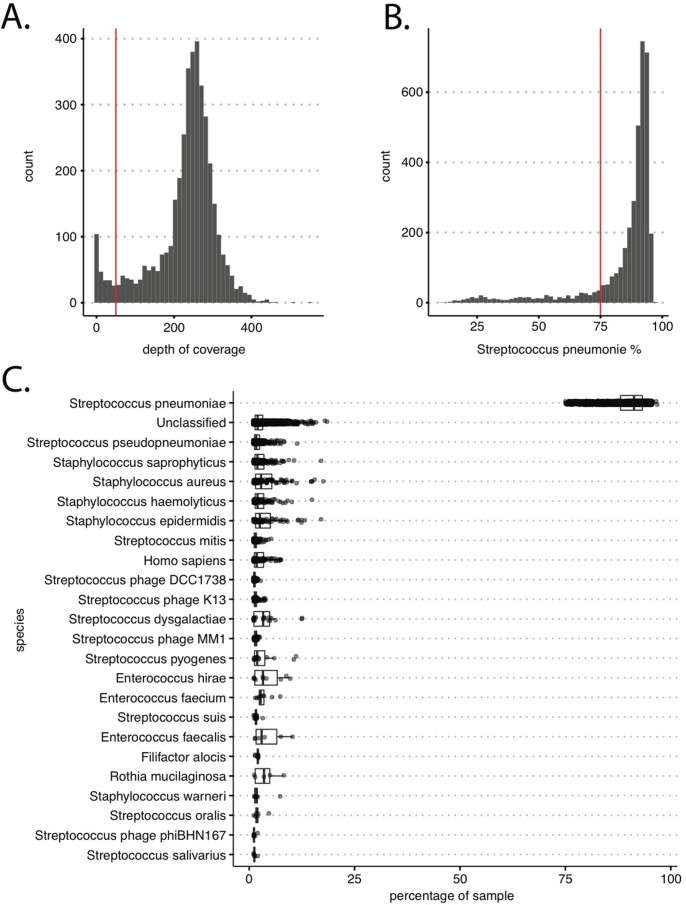
Distribution of sequencing coverage and contamination used to determine quality control cut-offs. The distribution of the depth of sequencing coverage (**a**) and fraction of reads (**b**) that aligned to *S. pneumoniae* using the Kraken2 metagenomics read classification algorithm. The vertical red lines indicate the minimum thresholds chosen for samples to be included in the main analysis. (**c**) Boxplots indicating the distribution of the fraction of reads assigned to each species in each of the 3761 samples by the Kraken2 metagenomics read classification algorithm. Due to the large sequence diversity within, and similarity between, *S. pneumoniae* and *S. pseudopneumoniae*, a large fraction of reads assigned as ‘unclassified’ and as *S. pseudopneumoniae* may actually belong to *S. pneumoniae* genomes. The median and interquartile range is given by the horizontal lines with the whiskers indicating the largest and smallest values excluding those outside 1.5 times the interquartile range.

In total, 3,961 samples were successfully sequenced, including 200 that were sequenced in replicate. To concentrate our efforts on those samples with sufficient data to retrieve reliable results, we excluded samples with a mean coverage below 50-fold, representing 20% of the median coverage observed across all samples (Extended Data Fig. 8a). While it is hard to choose an optimal coverage threshold, 50× has been shown to be a reasonable coverage for the assembly of bacterial genomes^65^.

To account for contamination from other species, Kraken (1.1.1) was run on all samples, with a histogram of the proportion of each sample assigned to *S. pneumoniae* given in Extended Data Fig. 8b. A threshold of requiring that at least 75% of reads were classified as *S. pneumoniae* was chosen as a compromise between avoiding excluding too many samples and ensuring that contamination did not bias our analyses. Further checks were also conducted at each stage of the downstream analyses to ensure results were not impacted by remaining low levels of contaminating species. Overall, this resulted in 3,188 samples including 164 replicates that were considered in the subsequent analysis steps.

### Lineage deconvolution

Lineage deconvolution was performed via the mSWEEP (v1.4.0) and mGEMS (v1.0.0) algorithms^66,67^ using a reference database consisting of a high-quality subset of 20,047 genomes from the Global Pneumococcal Sequencing Project database^9^. Included in this subset were 2,663 genome assemblies from the original genome sequencing study of the Maela camp that relied on single colony picks^21^. The PopPUNK algorithm, which uses a *k*-mer-based approach to cluster genomes into major lineages, was used to assign each of these genomes to their respective global pneumococcal sequencing cluster^68^. The mSWEEP and mGEMS pipelines were then run using the fastq files for each deep-sequencing sample, with the exact commands given in the Rmarkdown provided as part of the accompanying GitHub repository. The mSWEEP algorithm uses read pseudoalignments output by Themisto (v0.2.0) to quickly estimate the abundance of reference groups within a mixed sample using a statistical mixture model. mGEMS uses the resulting likelihood estimates output by mSWEEP to deconvolute the mixed reads into one or more groups. Importantly, reads may be assigned to multiple reference groups, accounting for the considerable homology between pneumococcal lineages. To reduce the possibility of false positives, lineages were only called if they were present at a frequency of at least 1%. The Mash Screen algorithm (v2.2.2), which similar to PopPUNK uses *k*-mers to assign reads to a reference database, was also run on each of the deconvoluted lineages using the same database^69^. Only lineages that shared at least 990/1,000 hashes were retained.

### Serotype calling

**Extended Data Fig. 9:**
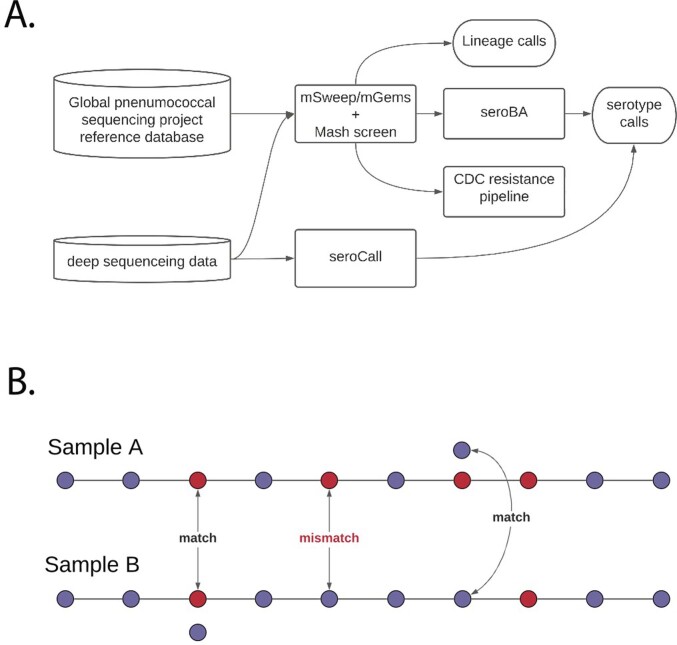
Schematics indicating the bioinformatics pipelines used to call serotypes, resistance elements and genetic distance in transmission calculations. (**a**) A schematic indicating the bioinformatics pipeline used to call both serotypes and resistance elements from the PDS data. (**b**) A schematic indicating how the pairwise SNP distance is calculated to account for within-host diversity and polymorphisms. Here, the red and blue indicate distinct nucleotides. A mismatch is only called if no alleles match at that location between the two samples. Variable sequencing coverage is accounted for using an empirical Bayes approach that made use of the multinomial Dirichlet distribution (methods).

Serotypes were identified by taking the union of two pipelines (Extended Data Figs. 1e and 9a). The serocall (v1.0) algorithm was run on the raw fastq files for each sample^22^. As a second step, the seroBA (v1.0.2) algorithm was run on each of the deconvoluted lineages identified by mGEMS pipeline^70^. By comparing the results of these pipelines on both artificial laboratory mixtures^22^ and samples for which single colony picks had also been performed, we were able to determine that while both algorithms generally agreed at the serogroup level, the serocall algorithm was more sensitive and was able to detect lineages below the 1% cut-off used in running mGEMS. As the serocall algorithm was less precise at distinguishing serotypes at the sub-group level (Extended Data Fig. 1d), whenever the pipelines produced conflicting results at the sub-serogroup level, the seroBA result was chosen. After taking the union of these two pipelines, we were able to correctly recover 93.6% of serotypes originally identified by latex sweeps performed on the same set of samples. The analysis of the artificial laboratory mixtures also indicated that the combined pipeline achieved a sensitivity of 0.93 with a precision of 1.

### Resistance calling

Similar to the calling of serotypes, resistance determinants were identified via two pipelines using the raw data and the deconvoluted output of the mGEMS pipeline. The pneumococcal-specific CDC resistance calling pipeline was run on each of the deconvoluted lineages identified using mGEMS^50^. This makes use of a database of PBP proteins with known resistance profiles. The combined mGEMS and resistance calling pipeline was found to achieve a sensitivity of 0.75 and precision of 0.825 in identifying resistance calls from the artificial laboratory mixtures. The lower accuracy in identifying resistance was caused by small inaccuracies in the deconvolution of strains and a lower sensitivity in detecting resistance in the sample containing 10 lineages. As the maximum number of lineages observed in any sample in our dataset was six, this drop in sensitivity at very high multiplicities of infection was not of concern. To account for inaccuracies in the deconvolution of resistance-associated sequencing reads, we only report resistance calls at the sample level. After restricting the comparison of laboratory calls to those samples continuing <10 lineages, we achieved an accuracy of 1 at the sample level. To verify the pipeline on a more diverse dataset, we compared the resistance calls found in 1,158 samples for which both single colony picks and whole-plate sweeps had been taken. The mGEMS + CDC pipeline was able to achieve a recall rate of 96.9%, indicating that the combined pipeline can accurately identify resistance from deep-sequenced plate sweeps. To check that the pipeline did not result in a high number of false positives, we compared the calls from single colony picks and plate sweeps on the subset of 584 samples that involve only a single lineage. Here we would expect the results of both approaches to be similar. Extended Data Fig. 2a indicates that there was no significant difference on this subset of samples, with only a very small increase of 2.7% (53/1,980) of resistance calls (*P* = 0.4, Poisson generalised linear model).

### Resistance co-occurrence

To examine whether certain lineages or serotypes were more likely to be found in instances of multiple colonization, we performed a logistic regression using a generalized linear mixed model with a complementary log-log link function. Lineages were classified as ‘resistant’ to each antibiotic class using the pneumococcal CDC resistance calling pipeline^48^. To control for the increase in the probability of resistance being present in a sample with multiple lineages simply because there were more lineages present, we used an offset term. This is a common approach used in ecological studies to control for the differences in exposure when investigating a binary outcome. This allows us to test whether the presence of resistance as a binary dependent variable is associated with multiple colonization beyond what would be expected given the background frequency of resistance in the population.

To control for the lineages present within each sample, we performed multidimensional scaling on a pairwise distance matrix inferred using the Mash algorithm^71^. The first ten components were included in the regression to control for population structure, as is common in bacterial GWAS studies^72^. Host effects were controlled for by including a random effect for the host.

### Genome-wide association analyses

To better account for the extensive pangenome in *S. pneumoniae*, locus-level association analyses were performed using an alignment-free method which first identifies all unique unitigs (variable length *k*-mers) within the samples being considered. Unitigs have been shown to better account for the diverse pangenomes found in bacteria^73^. The frequency of each unitig in each sample was obtained by first running the Bifrost algorithm to define the complete set of unitigs present^74^. The count of each unitig in each sample was then obtained using a custom Python script available in the accompanying GitHub repository. To avoid testing very rare features, we only considered those unitigs present in at least 1% of the samples of interest in our presence/absence-based analysis and in at least 2% of our paired analysis discussed below.

To investigate the impact of antibiotic treatment on *S. pneumoniae* carriage, we performed two main analyses. The first consisted of a typical case control design and compared samples that were within a recent antimicrobial treatment event to those where no treatment had occurred. This allowed us to investigate features associated with recent antibiotic treatment but does not consider the changes that occur within an individual that is already colonized with *S. pneumoniae* before treatment. To shed light on this scenario, our second analysis investigated the impact of treatment on pairs of consecutive samples from the same patient, where a subset of patients had received antibiotic treatment between samples (Extended Data Fig. 7a).

### Standard design

Samples were classified as treated if they were within 28 d of an antimicrobial treatment event. This was chosen after reviewing the decline in the proportion of resistant isolates tested via disk diffusion and Etest minimum inhibitory concentration (MIC) testing of all swabs positive for *S. pneumoniae* (Extended Data Fig. 7b). The Python implementation of the Seer algorithm was then used to identify unitigs significantly associated with treatment^75^. Here, rather than using counts, unitigs were called as either present or absent. To control for population structure, Pyseer (v1.3.9) was run using a linear mixed model, with a kinship matrix generated by taking the cross product of the binary unitig presence/absence matrix. Unitigs found to be significant were then aligned to a collection of pneumococcal reference genomes including all the single-genome assemblies of ref. ^21^, and assigned a gene annotation on the basis of the reference gene in which they aligned. Only those unitigs that were successfully aligned were considered for further analysis. To account for the large number of tests performed, we considered three *P*-value thresholds corresponding to an expected number of false discoveries (EFD) of 0.1, 1 and 5. The 0.1 threshold corresponds with the commonly used Bonferroni method, while the more relaxed thresholds allowed us to consider weaker signals. All three thresholds were more stringent than controlling for the false discovery rate using *q*-values which has been suggested as an alternative to the Bonferroni method as it is often found to be overly conservative^76^. Combined with past knowledge of possible resistance elements in *S. pneumoniae*, we were able to confidently identify associations.

### Paired design

Our unique sampling allows us to compare samples from the same individual before and after treatment. We first identified sample pairs where there were at most 100 d separating pneumococcal positive nasopharyngeal swabs from the same individual. We restricted our analysis to infants as treatment information for mothers was not available. To ensure that previous treatments before the first sample of an individual were not confounding our results, we excluded pairs with any treatment event within 28 d of the first swab. This resulted in 615 sets of paired samples. We classified these pairs into treated and untreated groups on the basis of whether or not the individual had received antibiotic treatment in the time between swabs. A treatment event was defined to include any antibiotic class, although amoxicillin made up the vast majority (66.9%). The prescription of antimicrobials in the study participants was monitored by the study team and care was taken to document both antimicrobials prescribed by the Shoklo Malaria Research Unit clinic and those obtained from other sources^20^.

We only considered paired samples where the infants were positive for *S. pneumoniae* in both samples. As a result, we are not considering the impact of antibiotic treatment on overall carriage rates but rather the differences in *S. pneumoniae* genomes pre and post antibiotic treatment. Using this paired design, we considered the impact of treatment both at the lineage (GPSC) level as well as the locus level. Unlike many previous bacterial GWAS studies which typically focused on the presence or absence of a feature, we considered the frequency of both lineages and loci within each sample. This improves our ability to identify more subtle changes that can be obscured by ignoring within-host diversity.

#### Lineage level

At the lineage level, we considered the estimated frequencies of each lineage obtained using the mSWEEP algorithm. We used a simple linear model to test whether treatment impacted the frequency of the second sample of a pair after controlling for the observed frequency in the first sample as well as the difference in time between the two samples.

#### Locus model

To investigate locus-level effects, we considered the frequency of each unitig in each sample. To control for lineage-level effects, we concentrated on pairs where the same lineage was present in both samples. This reduced the analysis to 445 pairs.

Unlike the lineage-level analysis where we used estimated frequencies, unitigs were represented by the number of times they were observed in the raw reads from each sample. This is a similar problem to that found in the analysis of RNA-seq datasets where the number of RNA reads aligned to a gene was used as a proxy for the expression of that gene. Using an approach similar to that commonly used in the analysis of RNA-seq data, we fit a linear model to the log unitig counts normalized by the number of reads sequenced in each sample. Similar to the commonly used analysis of covariance (ANCOVA) method for analysing pre and post treatment data, we used the pre-treatment count to control for the paired nature of the data. We also included a covariate to control for the time between when the samples were taken. Further explanation and the code used to run all the association analyses are available in the Supplementary Text included in the GitHub repository.

### Within-host variant calling

**Extended Data Fig. 10:**
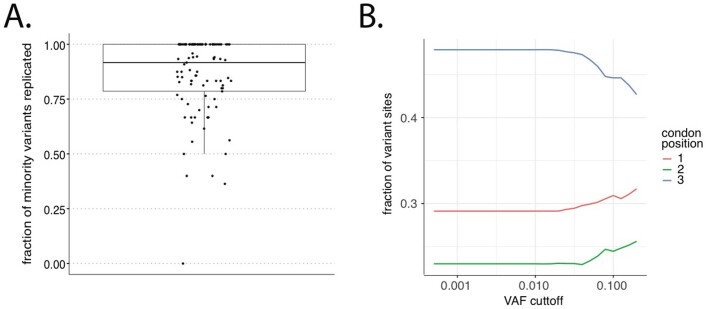
Reproducibility of single nucleotide (SNV) variant calls and the distribution of variable site among different coding positions used to assess the reliability of SNVs. (**a**) The fraction of minority single nucleotide variant calls replicated in 95 samples which involve only a single pneumococcal lineage and were sequenced in replicate with separate reverse transcription, PCR amplification, and library preparation steps. The median and interquartile range is given by the horizontal lines with the whiskers indicating the largest and smallest values excluding those outside 1.5 times the interquartile range (**b**). The distribution of the number of variable sites among different coding positions. Variable sites are dominated by those seen at the third codon position similar to that observed in Dyrdak et al., 2019. The stability of the fractions at lower frequencies suggests that the variant calling pipeline has successfully filtered out erroneous variant calls. At higher frequencies, the reduction in the total number of variants leads to increased variability.

To identify within-host variants, we ran the LoFreq (v2.1.5) variant calling pipeline on all samples for which only a single GPSC lineage had been identified with mSWEEP. The Lofreq pipeline has been shown to generate robust minority variant calls and accounts for base call qualities and alignment uncertainty to reduce the impact of sequencing errors^77^. To mitigate the impact of reference bias, each sample was aligned to a representative assembly (the medoid) for the GPSC that it most closely resembled via Mash distance^71^. Reads were aligned to the chosen reference genomes using BWA v0.7.17-r1188^78^. The Picard tools (v2.23.8) ‘CleanSam’ function was then used to soft clip reads aligned to the end of contigs and to set the alignment qualities of unaligned reads to zero. Pysamstats v1.1.2 was run to provide allele counts for each location of the aligned reference for use in the transmission analysis. The LoFreq pipeline was initially run with stricter filters, requiring a coverage of at least 10 reads to identify a variant. The resulting variant calls were used along with the read alignment as input to the GATK BaseRecalibrator tool (v4.1.9), as suggested in the LoFreq manual to improve the estimated base quality scores^79^. Finally, the LoFreq pipeline was run for a second time with a reduced coverage requirement of 3 reads. The resulting variant calls were only considered if there was support for the variant on at least two reads in both the positive and minus strand. In the remaining within-host single nucleotide variants, there was strong agreement between variant calls in the set of 95 sequencing replicates for which only a single lineage was present, with a median of 91.7% of variants recovered (Extended Data Fig. 10a). The distribution of minority variants among different coding positions was also consistent with real mutations rather than sequencing errors, with variants at the third codon position being most frequent (Extended Data Fig. 10b)^80^.

### Filtering problematic regions

To identify problematic variants that were probably the result of low-level contamination or multi-copy gene families, we implemented an approach similar to that used to identify recombination in the tool Gubbins^81^. A scan statistic was used to identify regions of the alignment with an elevated number of polymorphisms. Assuming that within-host variants are relatively rare and should be distributed fairly evenly across the genome, regions with a high number of polymorphisms are likely to be the result of confounding factors and can thus be filtered out.

We assumed a null hypothesis (*H*_0_) that the number of polymorphisms occurring in a window *s*_*w*_ follows a binomial distribution based on the number of bases within the window *w* and the mean density of polymorphisms across the whole alignment. We chose *w* for each sample such that *Expected*(*s*_*w*_) = 1. A window centred at each polymorphism was then considered and a one-tailed binomial test was performed to determine whether that window contained an elevated number of polymorphisms. After adjusting for multiple testing using the Benjamini-Hochberg method, windows with a *P* value <0.05 were selected and combined if they overlapped with another window^82^.

To define the edges of each region more accurately, we assumed that each combined window conformed to an alternative hypothesis *H*_1*r*_, where the number of polymorphisms *s*_*r*_ also followed a binomial distribution, with a rate based on the length of the window *l*_*r*_ and the number of polymorphisms within the window *s*_*r*_. Each end of the window was then progressively moved inward to the location of the next polymorphism until the likelihood of *H*_1*r*_ relative to *H*_0_ no longer increased. The resulting final windows were then called as potential problematic regions if they satisfied the inequality

$$\frac{{0.05}}{{g/l_f}} > 1 - \mathop {\sum}\limits_{i = 0}^{i = s_f - 1} {\left( {\begin{array}{*{20}{c}} {lf} \\ i \end{array}} \right)} d_0^i\left( {1 - d_0} \right)l_f - i$$

where *l*_*f*_ is the length of the final window, *g* is the length of the reference genome and *d*_0_ is the expected rate of polymorphisms under the null hypothesis. The left-hand side of the equation accounts for the possible number of similarly sized non-overlapping windows in the reference. To further reduce the chance that spurious alignments between homologous genes could bias our results, we took a conservative approach and excluded mutations that were found within a single read length (150 bp).

### Mutational spectrum

In the mutational spectrum analysis of human cancers, normal samples are usually taken along with samples of the cancer to allow for somatic mutations to be distinguished from germline mutations. As we cannot be sure which alleles were present at the start of a pneumococcal carriage episode, we cannot be certain of the direction a mutation occurred in. For example, it is difficult to distinguish between an A→C and a C→A mutation. Instead, we considered the difference between the consensus and minority variants at each site in the reference genome. If we assume that the colonizing variant typically dominates the diversity within an infection, then this approach corresponds with the direction of mutation. To account for the context of each mutation, we considered the consensus nucleotide bases on either side of the mutation. These were then normalized to account for the overall composition of the reference genome for each GPSC. The normalized mutation rates (*r*) for each of the 192 possible changes (*j*) in a trinucleotide context were calculated as:

$$r_j = \frac{{n_j}}{{L_j\mathop {\sum}\nolimits_j {\frac{{n_j}}{{L_j}}} }}$$

where *n*_*j*_ is the total number of mutations observed for a trinucleotide change *j*, and *L*_*j*_ is the total number of times that the corresponding trinucleotide is present in the reference genome. To avoid double counting the same mutation, each variant was only counted once per host. The resulting frequencies for within and between hosts are given in Extended Data Fig. 4. The frequencies of each of the single nucleotide changes without accounting for sequence context were calculated similarly.

To compare with the mutational spectrum observed across a longer timescale, we considered the recombination-filtered alignments of 7 major sequence clusters generated in the original publication of the single colony pick analysis of the Maela dataset^21^. We used Iqtree v2.1.2 to build a maximum-likelihood phylogeny for each alignment using a General Time Reversible model with 4 rate categories and enabled the ‘ancestral’ option to reconstruct the sequences at the internal nodes of the resulting phylogeny^83^. Mutations were called by considering changes in alleles between consecutive nodes of the phylogeny, and the mutational spectrum was normalized using the trinucleotide frequencies in the reconstructed ancestral sequence of the root node. A permutation test was used to compare the proportion of each mutation type found in the within-host and between-host sets.

### Selection

Selection analyses were performed using a modified version of the dNdScv package^40^ to allow for the incorporation of variants called against multiple reference genomes. Distinct from traditional approaches to estimating dN/dS ratios that were developed to investigate selection in diverse sequences and rely on Markov-chain codon substitution models, dNdScv was developed to compare closely related genomes such as those found in somatic mutation studies where observed changes often represent individual mutation events. dNdScv uses a Poisson framework allowing for more complex substitution models that account for context dependence and the non-equilibrium of substitutions in estimating dN/dS ratios^40^. This is particularly important in the case of sparse mutations in low-recombination environments, as is the case in pneumococcal carriage over short timescales. To avoid false signals of negative or positive selection that have been observed under simpler models^40^, dNdScv uses a Poisson framework to account for the context dependence of mutations and non-equilibrium sequence composition, and to provide separate estimates of dN/dS ratios for missense and nonsense mutations.

To extend dNdScv to allow for the use of multiple reference genomes, we first clustered the gene regions from the annotated reference genomes using Panaroo v1.2^84^. The impact of each of the mutations identified using the LoFreq pipeline was inferred with dNdScv for each sample separately, using the corresponding reference genome and gene annotation file. The combined calls for each orthologous cluster were then collated and the collated set used to infer genome-wide and gene-level dN/dS estimates using a modified version of dNdScv available via the GitHub repository that accompanies this manuscript. We used the default substitution model in dNdScv, which uses 192 rate parameters to model all possible mutations in both trends in a trinucleotide contact as well as two *w* parameters to estimate the dN/dS ratios for missense and nonsense mutations separately. Due to the large number of samples, we used the more conservative dNdSloc method which estimates the local mutation rate for a gene from the synonymous mutations observed exclusively within that gene^85^. Care is needed when interpreting dN/dS ratios estimated from polymorphism data as they can be both time dependent, providing weaker signals of selection for more recent changes, and can be biased by the impacts of recombination^86^. However, these are unlikely to have caused substantial issues in this analysis as the short timescales involved mean that recombination was unlikely to have occurred at a rate sufficient to bias the results and as each variant call was derived at the sample level rather than by the comparison of two separate samples, as is typically the case in dN/dS studies relying on multiple sequence alignments of diverse sequences. As an extra precaution, we also excluded gene clusters identified as paralogous by the Panaroo algorithm to reduce the chance that spurious alignments between paralogous genes could bias the results.

### Transmission inference

To identify the likelihood of transmission between each pair of hosts, we extended the TransCluster algorithm to account for genetic diversity within the host and to be robust to deep-sequencing data involving multiple lineages.

The TransCluster algorithm expands the commonly used approach of using an SNP distance threshold to exclude the possibility of direct transmission to account for both the date of sampling and the estimated epidemiological generation time of the pathogen^30^. However, hypermutating sites, contamination, sequencing error, multi-copy gene families and multiple colonization all present additional challenges when investigating transmission using within-host diversity information^15,41^.

To account for these challenges, we took a conservative approach and estimated the minimum pairwise SNP distance that could separate any pair of genomes taken from two samples. Thus, two samples were only found to differ at a site if none of the alleles in either sample at that site were the same (Extended Data Fig. 9b). To allow for variation in sequencing depth across the genome, we used an empirical Bayes approach to provide pseudocounts for each allele at each site, informed by the allele frequency distribution observed across all sites. A multinomial Dirichlet distribution was independently fit to the allele counts for each sample via the maximum-likelihood fixed-point iteration method. The inferred parameters were then used as pseudocounts and a frequency cut-off corresponding to filtering out variants less than 2% was used. All variant calls that were observed were retained. This approach provides a lower-bound estimate of the genetic divergence separating any pair of pneumococcal genomes within each of the two samples while allowing for the possibility of multiple colonization (see Supplementary GitHub repository).

The estimated minimum SNP distance was then used as input to the TransCluster algorithm, assuming a mutation rate of 5.3 SNPs per genome per year and a generation time of 2 months. These values were inferred using an adapted version of the TransPhylo algorithm on the previously sequenced single colony picks from the Maela camp (see Supplementary Methods included in the accompanying GitHub repository)^21^. The estimated substitution rate conforms with previous studies investigating short-term evolutionary rates in *S. pneumoniae*^14^ and the estimated generation time is consistent with previous estimates of pneumococcal carriage durations and a uniform distribution of transmission events^44^. This resulted in estimates of the most probable number of intermediate hosts separating two sequenced pneumococcal samples. These estimates were then combined with epidemiological and serological information to identify the most probable direction of transmission between mothers and their children, as is described in the main text.

To investigate the transmission bottleneck, we compared the distribution of the number of shared polymorphic sites in samples with the most probable number of intermediate hosts, as inferred using the TransCluster algorithm (Fig. 2e). The effects of hypermutable sites, sequencing errors and multiple infections, which have been shown to confound efforts to estimate the size of the transmission bottleneck, are likely to be similar irrespective of how close two samples are in the transmission chain^41^. Thus, any increase in the number of shared polymorphic sites between samples that are likely to be related by recent transmission is probably the result of multiple genotypes being transmitted (Fig. 2e).

### Reporting summary

Further information on research design is available in the Nature Research Reporting Summary linked to this article.

## Supplementary information

Reporting Summary

Supplementary Tables 1 and 2 Accession codes for each sample used in this study along with the date the sample was collected and the corresponding lineage, serotype and resistance calls.

## Data Availability

Metadata originally collected in ref. ^20^ are available from https://github.com/gtonkinhill/pneumo_withinhost_manuscript. To protect the anonymity of study participants, some epidemiological data have been obscured in the publicly available files. The original metadata files are available on request via the MORU Tropical Health Network Data Access Committee https://www.tropmedres.ac/units/moru-bangkok/bioethics-engagement/data-sharing. Raw sequencing data are stored with the ENA under project code PRJEB22771, with individual accessions given in Supplementary Table 1. The following previously published datasets were used: ref. ^21^; NCBI Sequencing Read Archive, ERP000435, ERP000483, ERP000485, ERP000487, ERP000598 and ERP000599; Global Pneumococcal Sequencing project; ENA RJEB3084.

## Code availability

Supplementary code is available from https://github.com/gtonkinhill/pneumo_withinhost_manuscript. The transmission clustering implementation is available at https://github.com/gtonkinhill/fasttranscluster. The modified version of the dndscv algorithm is available at https://github.com/gtonkinhill/dndscv.
