# Supplementary material for: Pneumococcal within-host diversity during colonization, transmission and treatment
Source: Nat Microbiol. 2022 Oct 10;7(11):1791–804. doi: 10.1038/s41564-022-01238-1 (PMC9613479; doi:10.1038/s41564-022-01238-1)
Supplement: Supplementary file 1 — Reporting Summary [file 41564_2022_1238_MOESM1_ESM.pdf]

nature portfolio

Corresponding author(s): Gerry Tonkin-Hill, Stephen Bentley

Last updated by author(s): Jul 4, 2022

Reporting Summary

Nature Portfolio wishes to improve the reproducibility of the work that we publish. This form provides structure for consistency and transparency in reporting. For further information on Nature Portfolio policies, see our Editorial Policies and the Editorial Policy Checklist.

Statistics

For all statistical analyses, confirm that the following items are present in the figure legend, table legend, main text, or Methods section.

n/a	Confirmed	
☐	☒	The exact sample size (*n*) for each experimental group/condition, given as a discrete number and unit of measurement
☐	☒	A statement on whether measurements were taken from distinct samples or whether the same sample was measured repeatedly
☐	☒	The statistical test(s) used AND whether they are one- or two-sided<br>*Only common tests should be described solely by name; describe more complex techniques in the Methods section.*
☐	☒	A description of all covariates tested
☐	☒	A description of any assumptions or corrections, such as tests of normality and adjustment for multiple comparisons
☐	☒	A full description of the statistical parameters including central tendency (e.g. means) or other basic estimates (e.g. regression coefficient) AND variation (e.g. standard deviation) or associated estimates of uncertainty (e.g. confidence intervals)
☐	☒	For null hypothesis testing, the test statistic (e.g. *F*, *t*, *r*) with confidence intervals, effect sizes, degrees of freedom and *P* value noted<br>*Give P values as exact values whenever suitable.*
☒	☐	For Bayesian analysis, information on the choice of priors and Markov chain Monte Carlo settings
☒	☐	For hierarchical and complex designs, identification of the appropriate level for tests and full reporting of outcomes
☐	☒	Estimates of effect sizes (e.g. Cohen's *d*, Pearson's *r*), indicating how they were calculated

Our web collection on statistics for biologists contains articles on many of the points above.

Software and code

Policy information about availability of computer code

Data collection	No software was used for data collection
Data analysis	s All code and scripts used to analyse the data are publicly available at https://github.com/gtonkinhill/pneumo_withinhost_manuscript, https://<br>github.com/gtonkinhill/fasttranscluster and https://github.com/gtonkinhill/dndscv. Software packages used in this analysis include Themisto (v0.2.0-1), mSWEEP (v1.4.0), mGEMS (v1.0.0), Mash (v2.2.2), serocall (v1.0), seroBA (v1.0.2), Pyseer (v1.3.9), LoFreq (v2.1.5), BWA v0.7.17-r1188, Picard tools (v2.23.8), Pysamstats v1.1.2, BaseRecalibrator tool (v4.1.9), Iqtree (v2.1.2) , Panaroo (v1.2) and R (v3.6).

For manuscripts utilizing custom algorithms or software that are central to the research but not yet described in published literature, software must be made available to editors and reviewers. We strongly encourage code deposition in a community repository (e.g. GitHub). See the Nature Portfolio guidelines for submitting code & software for further information.

Data

Policy information about availability of data

All manuscripts must include a data availability statement. This statement should provide the following information, where applicable:

- Accession codes, unique identifiers, or web links for publicly available datasets
- A description of any restrictions on data availability
- For clinical datasets or third party data, please ensure that the statement adheres to our policy

Meta data is available from https://github.com/gtonkinhill/pneumo_withinhost_manuscript. To protect the anonymity of study participants some epidemiological data has been obscured in the publicly available files. The original metadata files are available on request via the MORU Tropical Health Network Data Access Committee https://www.tropmedres.ac/units/moru-bangkok/bioethics-engagement/data-sharing.
2
nature portfolio | reporting summary March 2021
Raw sequencing data is stored with the ENA under project code PRJEB22771 with individual accessions given in Supplementary Table 1.

The following previously published datasets were used:

Chewapreecha et al., 2014. NCBI Sequencing Read Archive ERP000435, ERP000483, ERP000485, ERP000487, ERP000598 and ERP000599.

Bentley SD. 2019. Global Pneumococcal Sequencing project. ENA. PRJEB3084

Human research participants

Policy information about studies involving human research participants and Sex and Gender in Research.

Reporting on sex and gender	Infants sex and gender are not reported and sex based analyses were not performed.
Population characteristics	Nasopharyngeal swabs were collected between November 2007 and November 2010 from an initial cohort of 999 pregnant women leading to the enrolment of 965 infants from the Maela refugee camp in Thailand.
Recruitment	Samples were taken from those originally collected as part of the Maela pneumococcal carriage study (Turner et al., 2012). Briefly the recruitment occurred between October 2007 and November 2008, when all pregnant women attending the SMRU antenatal clinic at 28–30 weeks gestation were invited to consent to their infant's participation in a pneumonia cohort study. Using sealed opaque envelopes containing an allocation code, women were randomly allocated to the pneumococcal carriage sub-cohort at enrolment. For this sub-cohort, women had a nasopharyngeal swab (NPS) taken at delivery and both infant and mother had a NPS taken at monthly surveillance visits from 1–24 months of age.
Ethics oversight	Faculty of Tropical Medicine, Mahidol University, Thailand (MUTM-2009-306) and Oxford University, UK (OXTREC-031-06)

Note that full information on the approval of the study protocol must also be provided in the manuscript.

Field-specific reporting

Please select the one below that is the best fit for your research. If you are not sure, read the appropriate sections before making your selection.

☒ Life sciences ☐ Behavioural & social sciences ☐ Ecological, evolutionary & environmental sciences

For a reference copy of the document with all sections, see nature.com/documents/nr-reporting-summary-flat.pdf

Life sciences study design

All studies must disclose on these points even when the disclosure is negative.

Sample size	No formal sample size calculation was performed. However, the sample size was chosen to be sufficiently large such that: all samples collected in the original study by Turner et al., 2011 that occurred before and after antimicrobial treatment were included; all samples found to be within 10 SNPs in the study of Chewapreecha et al., 2014 could be included and; samples with a resolution of at least one every 2 months could be included from a subset of 25 mother/child pairs. Culture and sequencing was attempted on a total of 4000 samples (including replicates) of which 3188 passed quality control checks.
Data exclusions	Only samples that failed initial quality control as described in the methods section of the manuscript were excluded from subsequent analyses.
Replication	To check for potential processing artifacts, 192 of the selected samples were sequenced in replicate with separate PCR amplification and library preparation steps. The culture step was also replicated in a further 8 samples of which 3 passed initial quality control filters. A further subset of 1158 the samples were separately cultured and single colony picks sequenced in the previous study of Chewapreecha et al., 2014.

Randomization	Samples taken within 2 months of a antimicrobial treatment event were allocated to the 'treated' group with the remaining samples allocated as 'untreated'. No further allocation into groups was done. Other covariates such as the person being samples, the timing of samples and duration of pneumococcal carriage were included as variables in the regression analyses.
Blinding	No blinding was performed. Antimicrobial treatment was given based on the health requirements of the infants as determined by a doctor and was not determined by this study.

Reporting for specific materials, systems and methods

We require information from authors about some types of materials, experimental systems and methods used in many studies. Here, indicate whether each material, system or method listed is relevant to your study. If you are not sure if a list item applies to your research, read the appropriate section before selecting a response.

Materials & experimental systems

n/a	Involved in the study
☒	☐ Antibodies
☒	☐ Eukaryotic cell lines
☒	☐ Palaeontology and archaeology
☒	☐ Animals and other organisms
☒	☐ Clinical data
☒	☐ Dual use research of concern

Methods

n/a	Involved in the study
☒	☐ ChIP-seq
☒	☐ Flow cytometry
☒	☐ MRI-based neuroimaging
